# YOLO-SCL: a lightweight detection model for citrus psyllid based on spatial channel interaction

**DOI:** 10.3389/fpls.2023.1276833

**Published:** 2023-10-27

**Authors:** Shilei Lyu, Xu Zhou, Zhen Li, Xueya Liu, Yicong Chen, Weibin Zeng

**Affiliations:** ^1^ College of Electronic Engineering, College of Artificial Intelligence, South China Agricultural University, Guangzhou, China; ^2^ Pazhou Lab, Guangzhou, China; ^3^ Division of Citrus Machinery, China Agriculture Research System of MOF and MARA, Guangzhou, China

**Keywords:** citrus psyllids, small target detection, YOLO, recursive gated convolution, lightweight, hyperparameter optimization

## Abstract

Efficient and accurate detection and providing early warning for citrus psyllids is crucial as they are the primary vector of citrus huanglongbing. In this study, we created a dataset comprising images of citrus psyllids in natural environments and proposed a lightweight detection model based on the spatial channel interaction. First, the YOLO-SCL model was based on the YOLOv5s architecture, which uses an efficient channel attention module to perform local channel attention on the inputs in the recursive gated convolutional modules to achieve a combination of global spatial and local channel interactions, improving the model’s ability to express the features of the critical regions of small targets. Second, the lightweight design of the 21st layer C3 module in the neck network of the YOLO-SCL model and the small target feature information were retained to the maximum extent by deleting the two convolutional layers, whereas the number of parameters was reduced to improve the detection accuracy of the model. Third, with the detection accuracy of the YOLO-SCL model as the objective function, the black widow optimization algorithm was used to optimize the hyperparameters of the YOLO-SCL model, and the iterative mechanism of swarm intelligence was used to further improve the model performance. The experimental results showed that the YOLO-SCL model achieved a mAP@0.5 of 97.07% for citrus psyllids, which was 1.18% higher than that achieved using conventional YOLOv5s model. Meanwhile, the number of parameters and computation amount of the YOLO-SCL model are 6.92 M and 15.5 GFlops, respectively, which are 14.25% and 2.52% lower than those of the conventional YOLOv5s model. In addition, after using the black widow optimization algorithm to optimize the hyperparameters, the mAP@0.5 of the YOLO-SCL model for citrus psyllid improved to 97.18%, making it more suitable for the natural environments in which citrus psyllids are to be detected. The experimental results showed that the YOLO-SCL model has good detection accuracy for citrus psyllids, and the model was ported to the Jetson AGX Xavier edge computing platform, with an average processing time of 38.8 ms for a single-frame image and a power consumption of 16.85 W. This study provides a new technological solution for the safety of citrus production.

## 1 Introduction

Citrus is one of the most popular fruits in the world with numerous economic benefits (Liu et al., 2019). However, citrus huanglongbing (HLB) is a highly transmissible citrus disease that is difficult to prevent and control. HLB often leads to orchard yield reduction or even extinction, thereby affecting the safe citrus production. HLB is caused by the bast parasitic gram-negative bacterium Candidatus Liberibacter asiaticus (CLas) (Ma et al., 2022). HLB has a latent period, and its early yellowing symptoms are similar to fruit tree deficiencies (Jiao, 2016), making it difficult to diagnose accurately by hand. Currently, HLB is primarily diagnosed using polymerase chain reaction (PCR) detection technology (Bao et al., 2020; Wang et al., 2022b), but the low content and nonuniform distribution of CLas in plants lead to false-negative results of conventional PCR detection (Liang et al., 2018). Some studies have used near-infrared spectroscopy (Liu et al., 2016), hyperspectral remote sensing (Lan et al., 2019; Yang et al., 2021), and laser-induced breakdown spectroscopy (Yang et al., 2022) to diagnose HLB by analyzing the phenotype of the diseased plants and the degree of elemental uptake by the plants. However, these methods are complex, time-consuming, and difficult to meet large-scale management needs of orchards. Unlike the above studies, which are essentially based on HLB detection, this study explores the use of machine vision technology for detecting the primary vector of HLB, i.e., citrus psyllid, from the perspective of pest detection, which can provide early warning for orchard prevention and control.

In recent years, researchers have used machine vision technology to conduct a series of studies in the field of orchard pest detection. Wang et al. (Wang et al., 2021c) implemented the classification and localization of candidate bounding boxes in a real-time citrus-pest detection system, which can quickly detect red spiders and aphids with a detection accuracy of 91.0% and 89.0%, respectively, and an average processing speed as low as 286 ms for single-frame images. Shi et al. (Shi et al., 2023) proposed an adaptive spatial feature fusion-based lightweight detection model for citrus pests in the Papilionidae family. They integrated multiple optimization methods such as an adaptive spatial feature fusion module and an efficient channel attention mechanism into the YOLOX model, and achieved a higher than 95.76% mAP0.5 and an improvement in inference speed of 29 FPS over YOLOv7-x. Khanramaki et al. (Khanramaki et al., 2021) proposed a deep learning–based integrated classifier for detecting three citrus pests and achieved a 99.04% classification accuracy by developing an integrated classifier to detect citrus leafminer, sooty mold, and pulvinaria by considering the diversity of classification network level, feature level, and data level. Peng et al. (Peng et al., 2022) proposed a lychee pest detection model based on a lightweight convolutional neural network ShuffleNetV2 model, using the SimAM attention mechanism, Hardswish activation function, and migration learning method; the model detected 13 lychee pests, including Tessaratoma papillosa, with an accuracy of 84.9%. Ye et al. (Ye et al., 2021) proposed a multifeature fusion-based method for litchi pest detection, using the median filtering method for feature extraction of pests. The detection of three problems, namely, Tessaratoma papillosa, leaf rollers, and Pyrops candelaria, was achieved with a 95.35% accuracy by training the backpropagation network. Pang et al. (Pang et al., 2022) proposed an improved YOLOv4 target detection model that detected seven orchard insect pests, including Gryllotalpidae, with an average accuracy of 92.86% and a detection time of 12.22 ms for a single-frame image by optimizing the Nelder–Mead simplex algorithm and training method, as well as augmenting the training data. Li et al. (Li et al., 2023) proposed a pest detection model for passion fruit orchards based on the YOLOv5 model, employing the convolutional block attention module and mix-up data enhancement algorithm to detect 12 insect pests, including Bactrocera dorsalis Hendel, with an average accuracy of 96.51% and detection time of 7.7 ms. Zhang et al. (Zhang et al., 2022) proposed an orchard pest detection model that combines the YOLOv5 model and GhostNet and can detect seven orchard pests, such as chrysomelids, with a 1.5% increase in mAP0.5compared to YOLOv5.

In summary, machine vision technology has good feasibility in detecting orchard pests. However, the existing technology only excels in pest classification, and the presence of missed detections and false positives in the detection of pests, particularly for citrus psyllids, which have tiny bodies at the millimeter level and pose challenges to the effectiveness of target detection algorithms. To address this issue, this study developed a dataset of citrus psyllids in a natural environments and proposed a lightweight detection model based on spatial channel interaction(YOLO-SCL). The main research work includes:

1. In the recursive gated convolutions (*ɡ_n_* Conv), an efficient channel attention module is used for the feature maps after channel mixing to enhance the interactions between the local channels, achieve the interplay between global spatial interactions and local channel interactions, improve the feature representation of the model for critical regions, and enhance the contextual semantic information.
2. The C3 module in the neck network is operated by deleting the convolutional layers to maximally retain the detailed feature information of the small targets and effectively improve the model detection performance.
3. The black widow optimization algorithm (BWOA) is used to optimize hyperparameters of the YOLO-SCL model, making the optimized hyperparameters more suitable for the citrus psyllids detection task, as the optimal settings of the model hyperparameters are not always the same in different studies.

This study is outlined as follows: Section 2 presents the dataset construction; Section 3 introduces the design associated with the YOLO-SCL model; Section 4 outlines the BWOA algorithm utilized for optimizing hyperparameters of the YOLO-SCL model; Section 5 provides a detailed analysis of experimental results; and finally, in Section 6, we discuss and conclude our study.

## 2 Dataset description

### 2.1 Background

**Figure 1:**
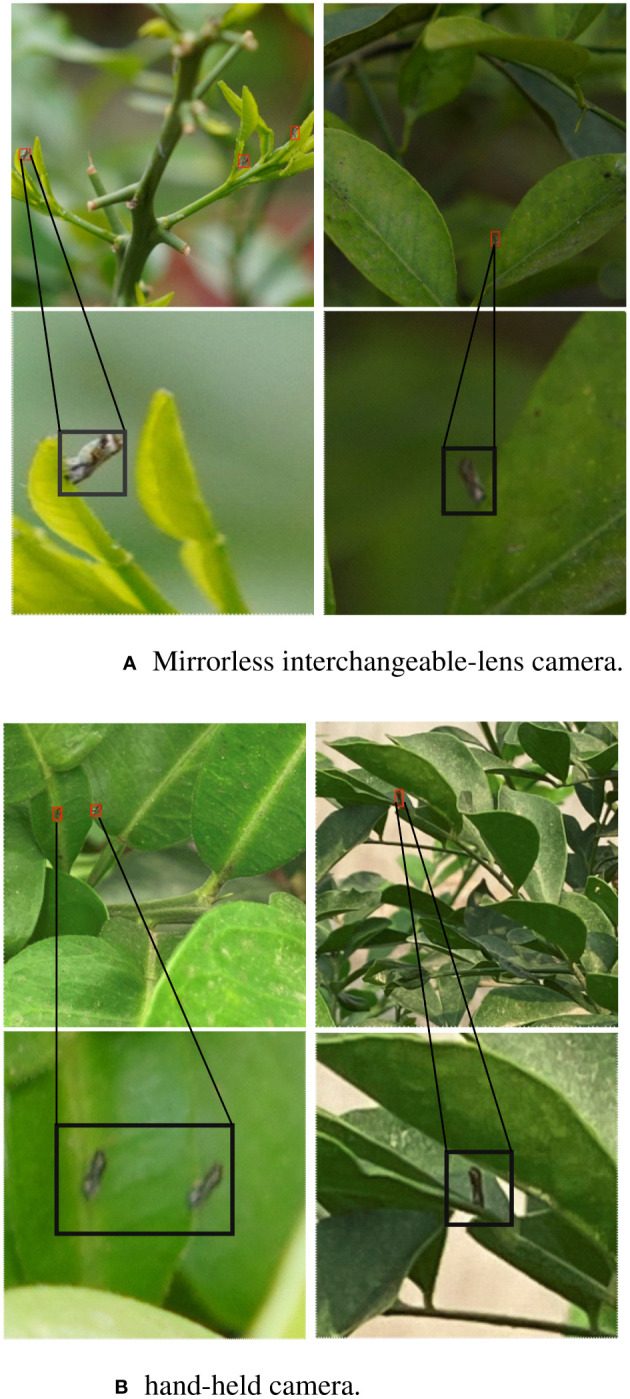
Example of citrus psyllid dataset. **(A)** Mirrorless interchangeable-lens camera. **(B)** hand-held camera.

The experimental data for this study were mainly collected from the experimental base of the citrus orchard of South China Agricultural University (Guangzhou, Guangdong, China), and the collection period was from June to December 2022. Considering that the phototropic behavior of adult psyllids is remarkably affected by light intensity (Yuan et al., 2020), the collection periods were primarily selected from 10:00 to 11:30 and 14:30 to 16:00 under natural light environment on sunny days, and the shooting tools included a mirrorless interchangeable-lens camera (SONY Alpha 6400 APS-C) and a hand-held camera (Xiaomi Mi 9, Honor 30), and the shooting distance was 50–100 cm. The image resolutions were 900 × 900 pixels (**Figure 1**).

### 2.2 Citrus psyllid dataset construction

The adult citrus psyllids was approximately 3 mm long, and the resolution of the psyllids in the captured images was less than 32 × 32 pixels, which was classified as a tiny target according to the target definition of the MS COCO dataset (Gao et al., 2021). The constructed citrus psyllid dataset has 2660 original images. The annotated dataset was categorized into the training (2,128 images) and validation sets (532 images) at a ratio of 8:2. The dataset was expanded to 7,980 images by adding salt-pepper noise, adjusting brightness, photoflipping, and other data enhancement methods. The dataset was annotated using the Labelimg tool, with 11,043 adult psyllid targets.

## 3 Lightweight YOLO-SCL detection model design

**Figure 2:**
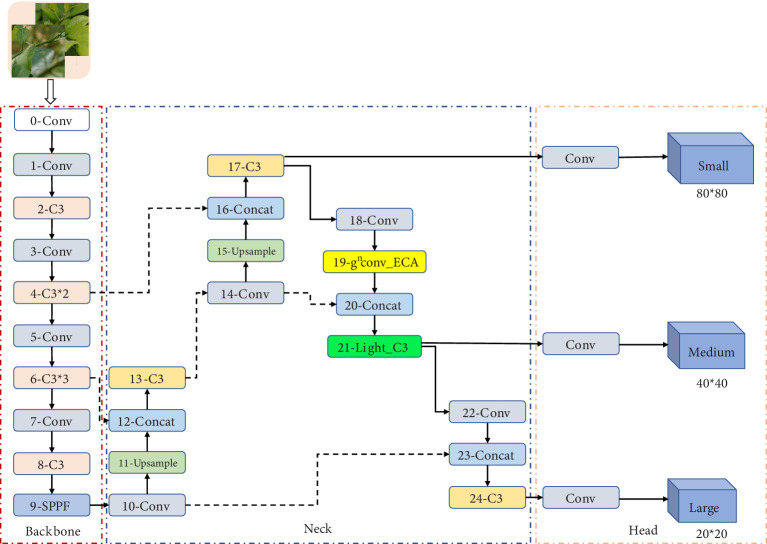
Schematic of citrus psyllid lightweight YOLO-SCL detection model.

**Figure 2** shows the overall flow of the proposed lightweight YOLO-SCL detection model for citrus psyllids, using YOLOv5 as the baseline model. First, an improved (*ɡ_n_* Conv) (19th layers) was added to the neck network to achieve long-distance modeling between features, improve feature representation in critical regions, and help the model resist complex background information. Second, the C3 module in the neck network was lightened to avoid too much loss of small target detail information while reducing the number of model parameters. **Table 1** shows the specific parameters of the YOLO-SCL model.

**Table 1:** YOLO-SCL model specific parameters table.

From	Params	Module	Arguments
-1	3520	Conv	[3, 32, 6, 2, 2]
-1	18560	Conv	[32, 64, 3, 2]
-1	18816	C3	[64, 64, 1]
-1	73984	Conv	[64, 128, 3, 2]
-1	115712	C3	[128, 128, 2]
-1	295424	Conv	[128, 256, 3, 2]
-1	625152	C3	[256, 256, 3]
-1	1180672	Conv	[256, 512, 3, 2]
-1	1182720	C3	[512, 512, 1]
-1	656896	SPPF	[512, 512, 5]
-1	131584	Conv	[512, 256, 1, 1]
-1	0	Upsample	–
[-1, 6]	0	Concat	[1]
-1	361984	C3	[512, 256, 1, False]
-1	33024	Conv	[256, 128, 1, 1]
-1	0	Upsample	–
[-1, 4]	0	Concat	[1]
-1	90880	C3	[256, 128, 1, False]
-1	147712	Conv	[128, 128, 3, 2]
-1	73059	*ɡ_n_* Conv ECA	[128]
[-1, 14]	0	Concat	[1]
-1	132096	Light C3	[256, 256, False]
-1	590336	Conv	[256, 256, 3, 2]
[-1, 10]	0	Concat	[1]
-1	1182720	C3	[512, 512, 1, False]

### 3.1 Improved recursive gated convolutions based on spatial channel interactions

**Figure 3:**
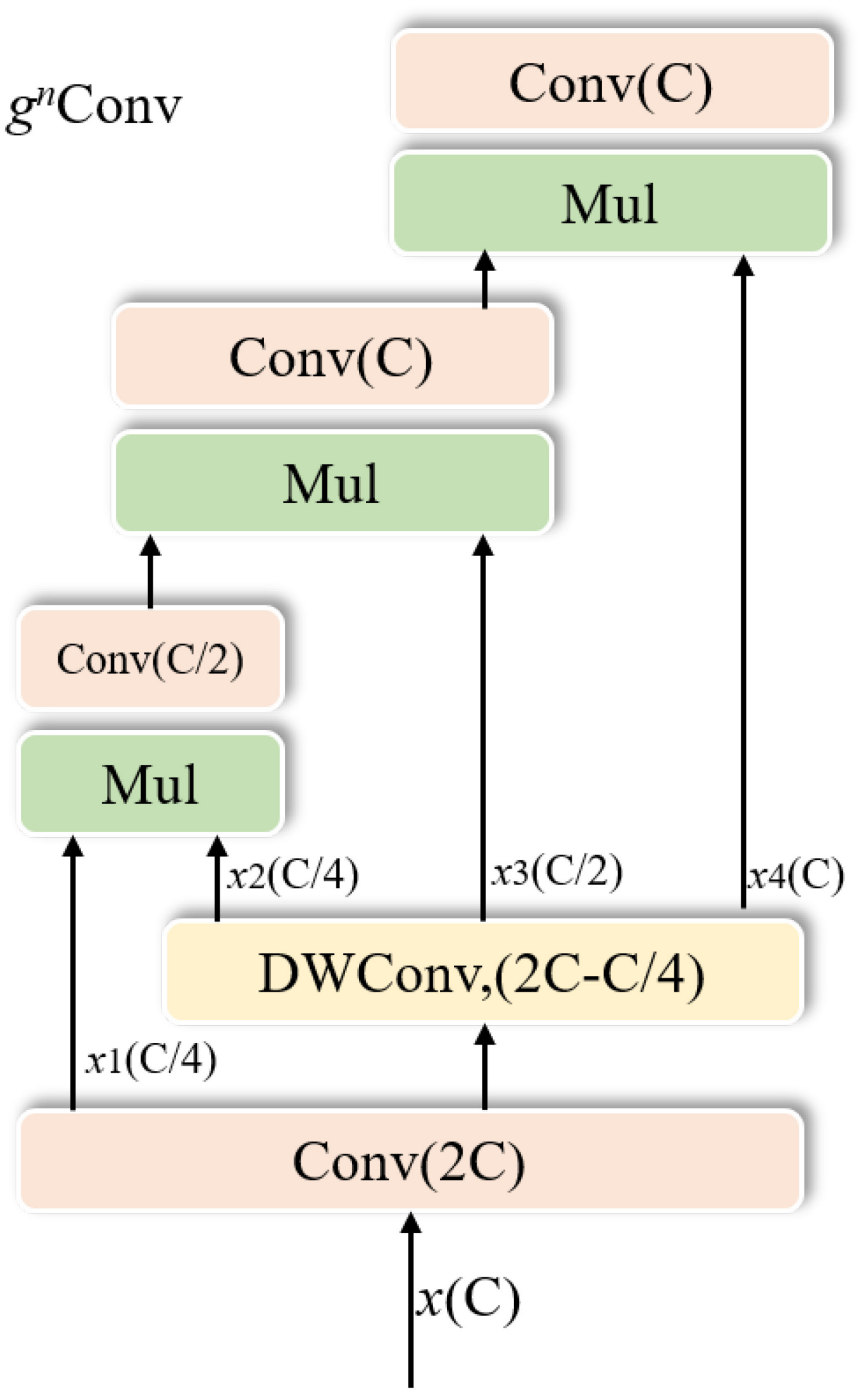
Recursive Gated Convolutions.

Transformer architecture was initially designed for natural language processing tasks (Vaswani et al., 2017). The vision transformer model (Dosovitskiy et al., 2020) was proposed to show that a vision model constructed only from transformer blocks and patch embedding layers can achieve performance comparable to those of convolutional neural networks. Unlike the convolutional kernel aggregation of the neighboring features approach, the visual transformer blends spatial elements using a multihead attention mechanism to achieve suitable modeling of spatial interactions in visual data. However, the quadratic complexity of the multihead self-attention mechanism remarkably limits its application, particularly in downstream tasks with high-resolution feature maps. The *ɡ_n_* Conv (Rao et al., 2022) implements spatial interactions using simple operations such as convolution and fully connected. Unlike the second-order interactions of the multiple attention mechanism, the *ɡ_n_* Conv module implements spatial interactions in an arbitrary order (**Figure 3**). The *ɡ_n_* Conv module enables the global spatial interaction through dot product operations, effectively overcoming the effect of complex backgrounds on target detection in citrus psyllids detection tasks. Therefore, in this study, we used the *ɡ_n_* Conv module and modified it for long-range feature modeling and feature representation in critical regions.

**Figure 4:**
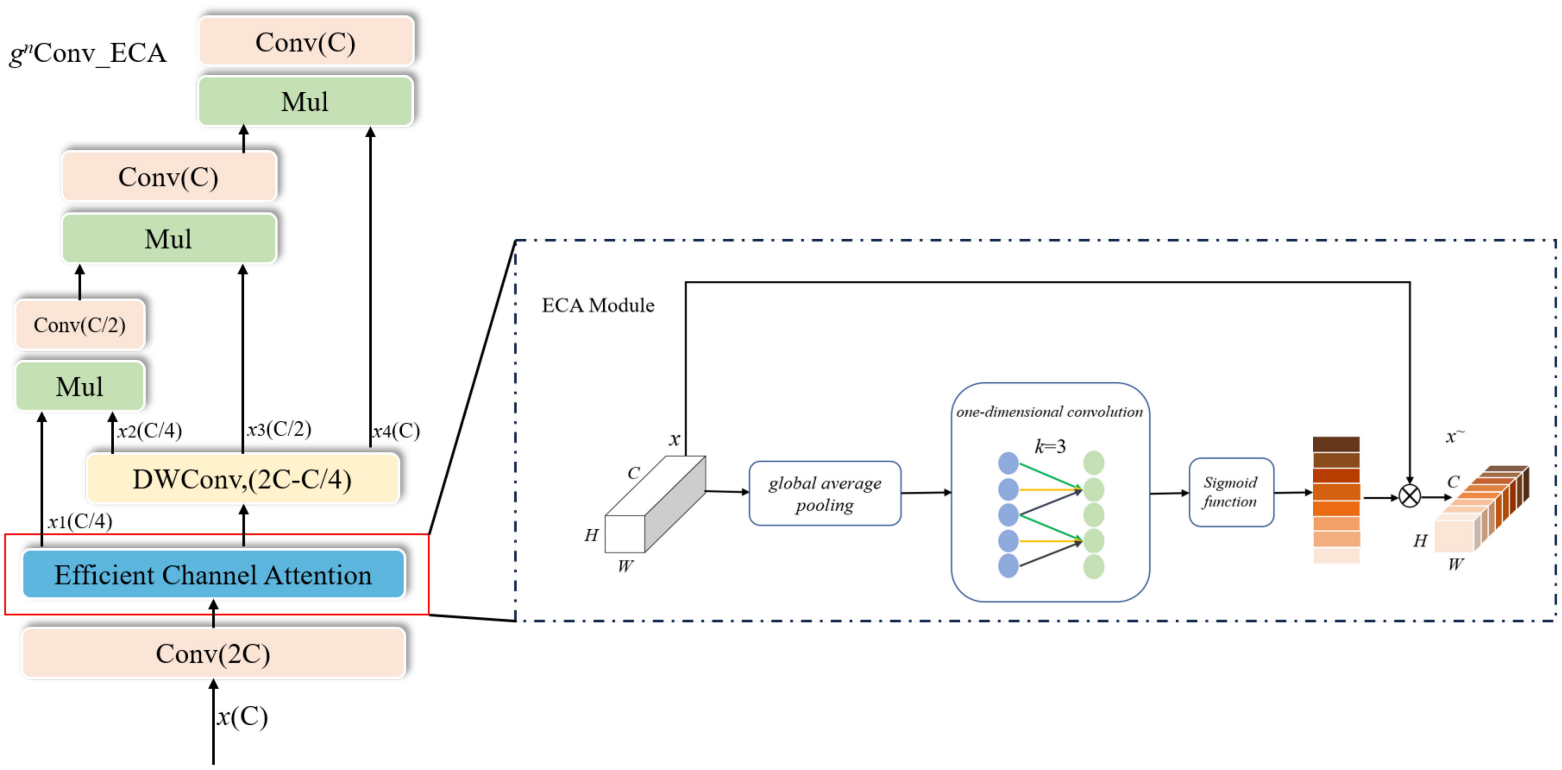
Improved Recursive Gated Convolutions based on spatial channel interaction.

The *ɡ_n_* Conv module implements global spatial interaction of features to establish long-range dependencies, but only performs simple channel blending of the input feature maps, ignoring interchannel interaction. While the channel attention mechanism can effectively improve the performance and screening features of deep learning networks, this study uses the channel attention module to enhance the *ɡ_n_* Conv module. Among the channel attention modules, the squeeze-and-excitation (SE) module (Hu et al., 2018) alters the feature dimensions, affecting the SE module’s performance. In contrast, the efficient channel attention (ECA) module (Wang et al., 2020) is a highly efficient and lightweight local channel interaction strategy that is improved on the SE module. The ECA module avoids feature dimensionality reduction and uses some parameters to achieve a significant performance improvement. In this study, the ECA module was added to the *ɡ_n_* Conv module (*ɡ_n_* Conv ECA module) (**Figure 4**). Using the ECA module to perform local channel interaction on feature maps after channel blending in the *ɡ_n_* Conv module improves the representation of the critical feature areas in the *ɡ_n_* Conv module.

The improved recursive gated convolution (*ɡ_n_* Conv ECA) module combines the global spatial and local channel interactions. The *ɡ_n_* Conv ECA module inputs feature maps in the following steps:

(1) First, a set of features *M*_0_ and *N_i_* are obtained by channel blending through a linear projection layer.


(1) $$\left[M_{0}^{HW\times C_{0}},N_{0}^{HW\times C_{0}},\ldots ,N_{n-1}^{HW\times C_{n-1}}\right]=\psi_{m}\left(x\right)$$

(2) This set of features is then passed through the ECA module to obtain a new set of features *M* ^+^_0_ and *N* ^+^ *_i_*.


(2) $$\left[M_{0}^{+HW\times C_{0}},N_{0}^{+HW\times C_{0}},\ldots ,N_{n-1}^{+HW\times C_{n-1}}\right]=ECA\left(\left[M_{0}^{HW\times C_{0}},N_{0}^{HW\times C_{0}},\ldots ,N_{n-1}^{HW\times C_{n-1}}\right]\right)$$

(3) Gated convolution is performed recursively using the following equation,


(3) $$M_{i+1}=DConv_{i}\left(N_{i}\right)⊙G_{i}\left(M_{i}\right)/S\;$$

(4) $$G_{i}=\{\begin{array}{cc} Identity, & i=0\\ Linear\left(C_{i-1},C_{i}\right), & 1\leq i\leq n-1 \end{array}$$

where *DConv_i_* is a set of deep convolutional layers, *G_i_* is the way of matching dimensions between different orders, and S is the scaling value to adjust the training stability. The formula for the ECA module in Eq. (2) is shown in Eq. (5).


(5) $${ⴘ}^{∼}=\sigma \left(\varphi \left(ⴘ\right)\right)⊗ⴘ$$

where *σ* is the Sigmoid activation function, *ϕ* is a 1 × k one-dimensional convolution, and y is the input feature map.

### 3.2 C3 module lightweight design

The main role of the convolutional layer is to extract image features. An appropriate increase in the number of convolution operations for images containing large targets can obtain richer semantic information, whereas in images containing small targets, successive convolution operations may cause too much loss of detail information of some of the small targets, leading to unsatisfactory results of the task, whereas in images containing small targets, successive convolution operations may cause too much loss of detail information of some of the small targets, leading to unsatisfactory results of the task.

**Figure 5:**
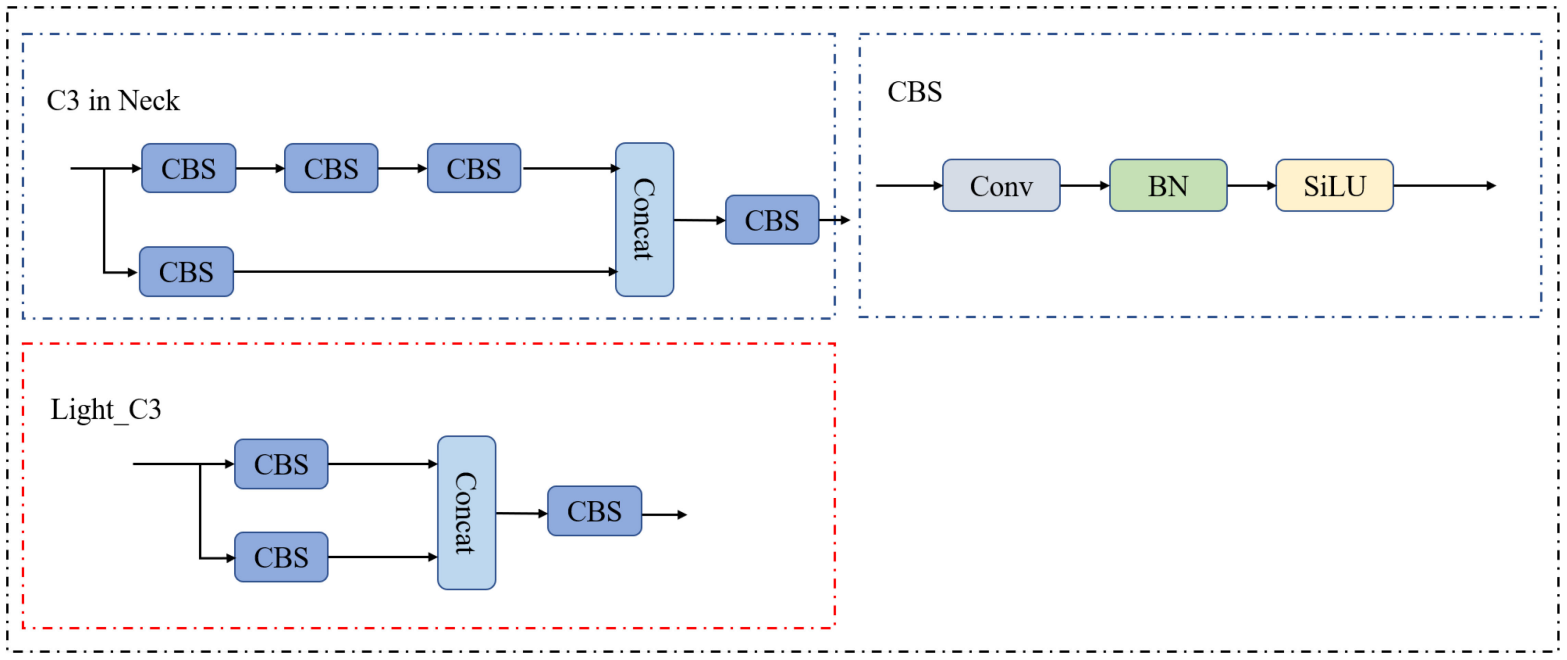
C3 module before and after improvement in the neck network.

In images with small targets, successive convolution operations may cause too much loss of detailed information on some of the small targets, leading to unsatisfactory task results. The citrus psyllids accounts for a tiny proportion of the image pixels, resulting in the loss of some of its detailed feature information as the number of network layers increases. In the neck network of the YOLO-SCL model, the 17th, 21st, and 24th layer C3 modules detect small, medium, and large targets, respectively. Based on the deepening of layers, their degree of retaining the detailed feature information of small targets decreases sequentially. Therefore, to retain more feature information of small targets, this study improved the 21st layer C3 module in the neck network of the YOLO-SCL model by deleting convolutional layers. **Figure 5** illustrates the C3 module before and after the improvement.

## 4 Hyperparameter optimization of the YOLO-SCL detection model

Considering the impact of hyperparameter settings on the performance of the detection model (Akay et al., 2022), this study adopts BWOA (F.Pena et al., 2020) to optimize the hyperparameters of the YOLO-SCL detection model. By leveraging the optimization iteration mechanism based on swarm intelligence, this study rapidly converged and obtained optimal hyperparameter combinations, thereby improving the performance of the model. This approach also addresses the issue of overreliance on experience in manual parameterization.

**Figure 6:**
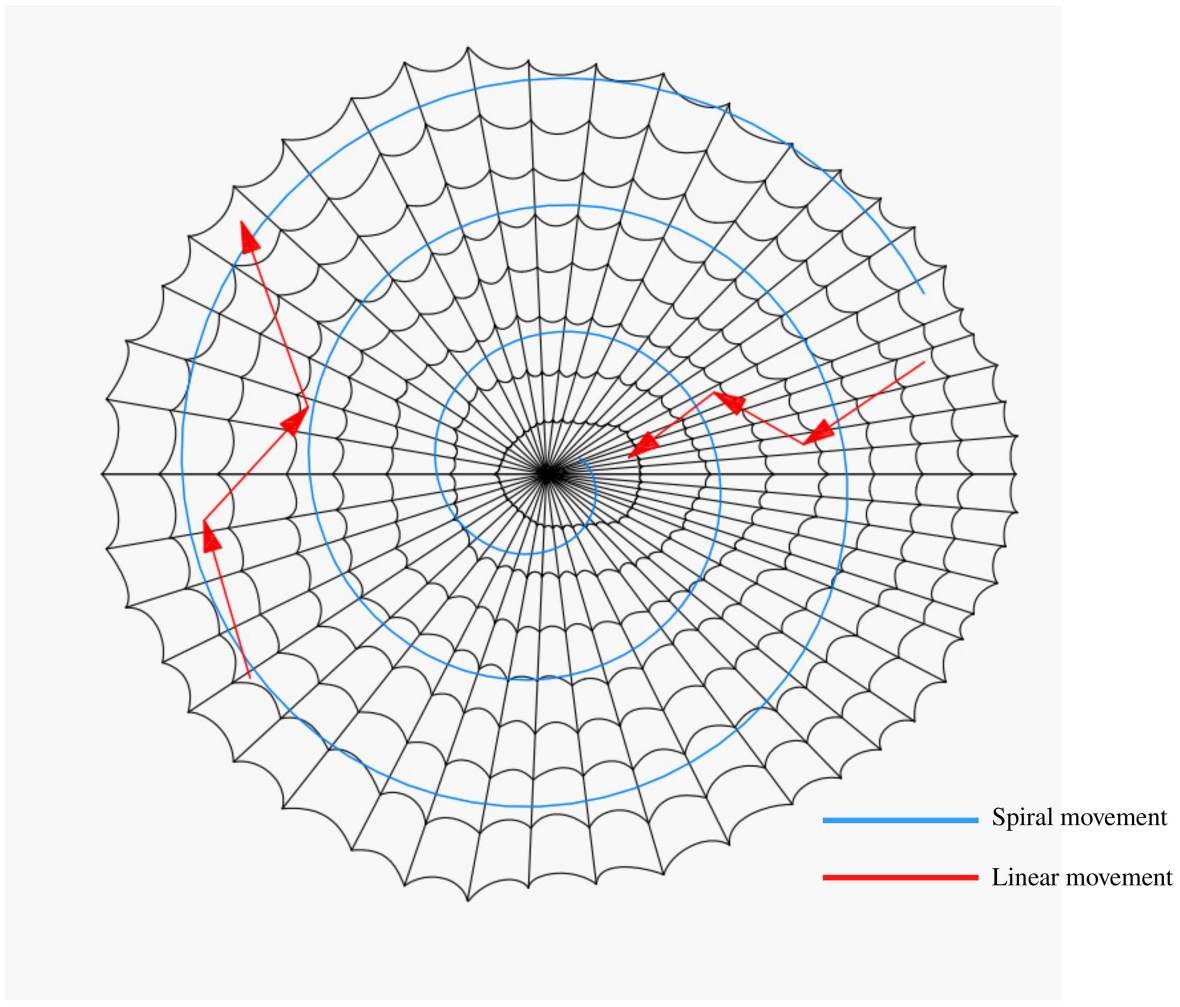
Black widow spider movement strategy.


BWOA is inspired by the behavior of black widow spiders, and the algorithmic model mainly consists of in-web movement and pheromone control strategies for black widow spider groups. **Figure 6** illustrates the movement strategy within the spider web, including both linear and spiral movement modes, which can be characterized by the formula shown in Eq. (6).

(6) $$x_{i}\left(t+1\right)=\{\begin{array}{cc} x_{gbest}\left(t\right)-mx_{r_{1}}\left(t\right), & if\;rand\leq 0.3\\ x_{gbest}\left(t\right)-\cos\;\left(2\pi \beta \right)x_{i}\left(t\right) & in\;\;other\;\;case \end{array}$$

where t is the current iteration number of the algorithm, *x_i_* (t) is the ith spider individual, *x_gbest_* (t) denotes the optimal spider individual of the current population, and *x_r_*_1_(t) denotes a randomly selected spider individual in the current population, *r*_l_ ≠ i. The control parameter m for the linear movement mode is a random floating-point number in an interval of [0.4,0.9], and the control parameter *β* for the spiral movement mode is a random floating-point number in an interval of [−1,1].

Second, the pheromone plays a crucial role in determining the state of individual spiders. During the mating process of male and female spiders, the spider that receives more nutrients exhibits better fecundity, resulting in a higher pheromone value that represents higher fecundity, which means higher fertility. The pheromone value for the ith spider individual in BWOA is calculated as shown in Eq. (7).


(7) $$phermone_{i}=\frac{fitness_{max}-fitness_{i}}{fitness_{max}-fitness_{min}}$$

where *fitness_i_* denotes the adaptation value of the ith spider individual, and *fitness_max_* and *fitness_min_* represent the worst and optimal adaptation values of the current population, respectively. Therefore, the range of pheromone values for spider individuals is [0,1]. In addition, if the pheromone value of spider individual i is not greater than 0.3, the position will be updated, as shown in Eq. (8).


(8) $$x_{i}\left(t\right)=x_{gbest}\left(t\right)+\frac{1}{2}\left[x_{r_{1}}-{\left(-1\right)}^{\sigma }\times x_{r_{2}}\left(t\right)\right]$$

where *x_r_*_1_(t), *x_r_*_2_(t) denote randomly selected spider individuals in the current population, *r*_1_≠ *r*_2_≠ i; *σ* takes values in the set {0,1}.

**Figure 7:**
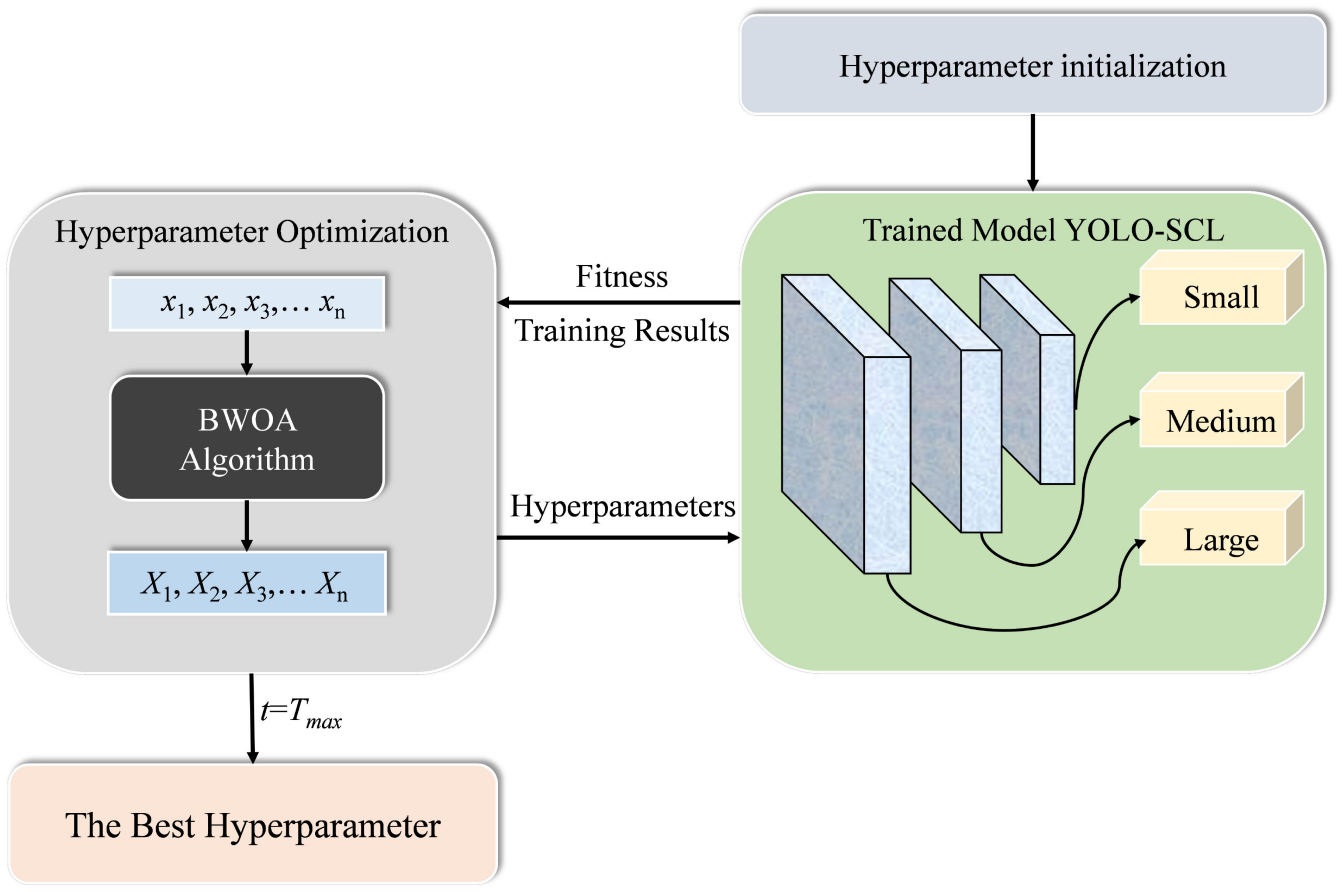
Flowchart of hyperparameter optimization of detection model based on BWOA algorithm.

**Figure 7** shows the flowchart of hyperparameter optimization of the detection model based on BWOA, and the computational steps include:

1) Data division: the dataset is collected and divided into training and validation sets.
2) Initialization of parameters and population: the parameters related to the algorithm are set, including the number of populations *N*, maximum number of iterations *T*, upper and lower bounds (*ub* and *lb*) of the hyperparameters, and the position of the population is initialized. Each individual represents a set of hyperparameters.
3) Calculation of fitness: after checking whether the position of an individual is out of bounds, each individual in the population is brought into the trained model to calculate the fitness value and each pheromone. The historical optimal individual *x_gbest_* is updated.
4) Hyperparameter optimization: each searching individual in the population updates the positional information according to the pheromone size using Eqs. (6) and (8), respectively, and updates the number of iterations (*t* = *t* + 1) and individual pheromones.
5) Algorithm termination: the algorithm terminates when the termination conditions are met, and the optimized hyperparameters *x_gbest_* are used to train the model and obtain the final model.

## 5 Experimental results and analysis

### 5.1 Experimental environment and model evaluation metrics

The YOLO-SCL model was created based on the deep learning framework pytoch1.12.0, and all model training was performed in Windows 10 with an Intel(R) Core(TM) i7-8700 CPU @ 3.20 GHz 3.19 GHz and a GPU of NVIDIA GeForce RTX 2080 Ti.

During training, each model uses stochastic gradient descent and cosine annealing to reduce the learning rate. To save computational resources, the epoch and batch sizes were set to 300 and 16, respectively, and an early stopping strategy was set to stop the training early if the model performance did not improve after 100 iterations.

In this study, typical evaluation metrics such as size, giga floating-point operations per second (GFlops), precision (P), recall (R), average precision (AP), and the mean average precision (mAP) were used to verify the performance of the target detection model. The calculation formulas are as follows.


(9) $$P=\frac{TP}{TP+FP}$$

(10) $$R=\frac{TP}{TP+FN}$$

(11) $$AP=\int_{0}^{1}P\left(R\right)dR$$

(12) $$mAP=\frac{1}{n}\left(\sum_{i=1}^{n}AP_{i}\right)$$

where true positives(TP) are the number of correctly detected targets, false positives (FP) are the number of samples in which annotation boxes were generated but in the wrong position or incorrectly labeled in the category, false negatives are the number of samples in which no annotation boxes were generated in the target. AP is equal to the area under the accuracy-recall curve, and mAP is the average of the AP in different categories. In this study, citrus psyllid is category 1, and the value of n is 1.

### 5.2 Benchmark model performance analysis

To achieve efficient and accurate detection of citrus psyllids small targets in complex environments, this study evaluated the performance of different YOLOv5 detection models based on the constructed citrus psyllid dataset. The experimental results are shown in **Table 2**. Among the different models tested, YOLOv5n had the lowest number of parameters and computations. However, its mAP@0.5 is lower than those of the YOLOv5s, YOLOv5m, YOLOv5l, and YOLOv5x models by 0.73%, 0.72%, 0.94%, and 1.16%, respectively. The mAP@0.5 of the YOLOv5m model is slightly lower than that of the YOLOv5s model, but the number of parameters and computations are approximately 2.97 and 3.03 times that of the YOLOv5s model. The mAP@0.5 of the YOLOv5l and YOLOv5x models are 0.21% and 0.43% higher than that of the YOLOv5s model, respectively. However, the number of parameters and computations amount of the YOLOv5l model is approximately 6.57 and 6.81 times that of the YOLOv5s model, and the number of parameters and computations amount of the YOLOv5x model is approximately 12.28 and 12.83 times more than the YOLOv5s model, respectively. Considering the number of parameters and computations, and mAP@0.5, YOLOv5s was chosen as the baseline model for the citrus psyllid detection task in this study.

**Table 2:** Detection results of different variants of the YOLOv5 model on the citrus psyllid dataset.

Model	Size (M)	GFlops	mAP@0.5 (%)
YOLOv5n	1.9	4.2	95.16
YOLOv5s	7.02	15.9	95.89
YOLOv5m	20.87	48.2	95.88
YOLOv5l	46.1	108.2	96.10
YOLOv5x	86.22	204.6	96.32

### 5.3 Analysis of the results of the ablation experiment

To achieve efficient and accurate detection of citrus psyllids small targets, ablation experiments were conducted on the constructed citrus psyllid dataset to analyze the importance of each module. The *ɡ_n_* Conv, *ɡ_n_* Conv ECA, and lightweight C3 (Light C3) modules were added to the model step-by-step to form several improved models, using the YOLOv5s model as the baseline model. The results of the experiments are shown in **Table 3**.

**Table 3:** Results of ablation experiments on the citrus psyllid dataset.

Methods	Baseline	*g_n_* Conv	*g_n_* Conv ECA	Light C3	Size (M)	GFlops	mAP @0.5 (%)	mAP@0.5:.95 (%)
YOLOv5s Model 1 Model 2 Model 3 Model 4	√ √ √ √ √	√	√ √	√ √	7.02 7.09 7.10 6.85 6.92	15.9 16.0 16.2 15.2 15.5	95.89 96.58 96.79 96.57 97.07	50.78 52.47 53.66 52.31 53.43

“√” that the component is selected for use into model YOLOv5.

**Table 3** shows that the model detection accuracy is improved by adding *ɡ_n_* Conv, *ɡ_n_* Conv ECA, and Light C3 modules. This is because the *ɡ_n_* Conv module implements global spatial interactions, constructing long-distance dependencies that better represent critical regions’ features. The *ɡ_n_* Conv ECA module combines global spatial interactions with local channel interactions to further improve the model’s ability to extract critical regions for accurate detection. The Light C3 module improves the C3 module by lightweight it, which retains the details of the small targets to a certain extent and achieves the purpose of reducing the number of model parameters, which helps to improve the model’s performance and reduce the amount of computation.

The mAP@0.5 and mAP@0.5:.95 of Model 1 (YOLOv5s+ *ɡ_n_* Conv model) and Model 2 (YOLOv5s+ *ɡ_n_* Conv ECA model) are improved by 0.69%, 0.90% and 1.69%, 2.88%, respectively, compared with the baseline model, which indicates that the global spatial interaction mechanism effectively improves the model’s detection of the target and that the *ɡ_n_* Conv ECA module incorporates the local channel interaction, which makes the target detection accuracy further enhanced. Model 3 (YOLOv5s+Light C3 model) not only has 1.70M fewer parameters than the baseline model but also has mAP@0.5 and mAP@0.5:.95 is 0.69% and 1.53% higher than the baseline model, indicating that the lightweight C3 module can effectively reduce the loss of the target feature information and ensure the improved detection accuracy while reducing the number of model parameters. Finally, the mAP@0.5 of model 4 (YOLOv5s+Light C3+ *ɡ_n_* Conv ECA model) reaches 97.07%, which is 1.18% higher than the baseline model, and both are higher than the other improved models.

### 5.4 Comparative analysis with other detection models

In this section, the proposed YOLO-SCL model is compared with mainstream single-stage target detection models, including the YOLOv4-CSP (Wang et al., 2021a), YOLOX (Ge et al., 2021), YOLOR-p6 (Wang et al., 2021b), YOLOv7 (Wang et al., 2022a), YOLOv8 (Reis et al., 2023) models, and transformer-based end-to-end detection model DETR (Carion et al., 2020). **Table 4** shows the test results, highlighting the advantage of YOLO-SCL model in terms of the number of parameters, computational load, and mAP compared to other control detection models. The improved architecture combining the *ɡ_n_* Conv and lightweight C3 modules effectively improves the feature representation of the model for target regions. This addresses the problem of the model losing semantic information for small targets as the layers deepen. The DETR model benefits from the transformer’s self-attention mechanism, and its mAP@0.5 is higher than that of the YOLOX and YOLOR-p6 models by 0.01% and 0.29%, respectively. However, compared with the YOLO-SCL model, the mAP@0.5 and mAP@0.5:.95 of the DETR model is lower than that of the YOLO model by 3.64% and 4.01%. The parameters and computations are 5.31 and 6.54 times higher than those of the YOLO-SCL model. The YOLOv4-CSP model, which is an improved model based on YOLOv4, obtained 94.56% mAP@0.5 and 50.41% mAP@0.5:.95 on the citrus psyllid detection task but was still 2.51% and 3.02% lower than the YOLO-SCL model. The YOLOv7 model mAP@0.5 is higher than other detection models. However, it is 1.23% lower than that of the YOLO-SCL model mAP@0.5 and 3.35% lower than that of the YOLO-SCL model mAP@0.5:.95, and the number of parameters and the amount of computation of the YOLOv7 model is much larger than that of the YOLO-SCL model. The YOLOv8 model mAP@0.5 is 1.86% lower than that of the YOLO-SCL model. However, it is 0.53% higher than the YOLO-SCL model mAP@0.5:.95. In summary, the proposed YOLO-SCL model has a parameter count of 6.92 M, which is easy to deploy on a mobile platform, and the model has the highest mAP@0.5, making it more suitable for the task of citrus psyllids detection in natural environments.

**Table 4:** Comparison of the performance of the latest models on the citrus psyllid dataset.

Methods	Size (M)	GFlops	mAP@0.5 (%)	mAP@0.5:.95 `(%)
YOLOv4-CSP	52.50	119.7	94.56	50.41
YOLOX	8.94	26.76	93.42	48.67
YOLOR-p6	36.8	80.6	93.14	49.46
YOLOv7	37.2	105.1	95.84	50.08
YOLOv8	11.2	28.6	95.21	53.96
DETR	36.74	101.4	93.43	49.42
YOLO-SCL	6.92	15.5	97.07	53.43

**Figure 8:**
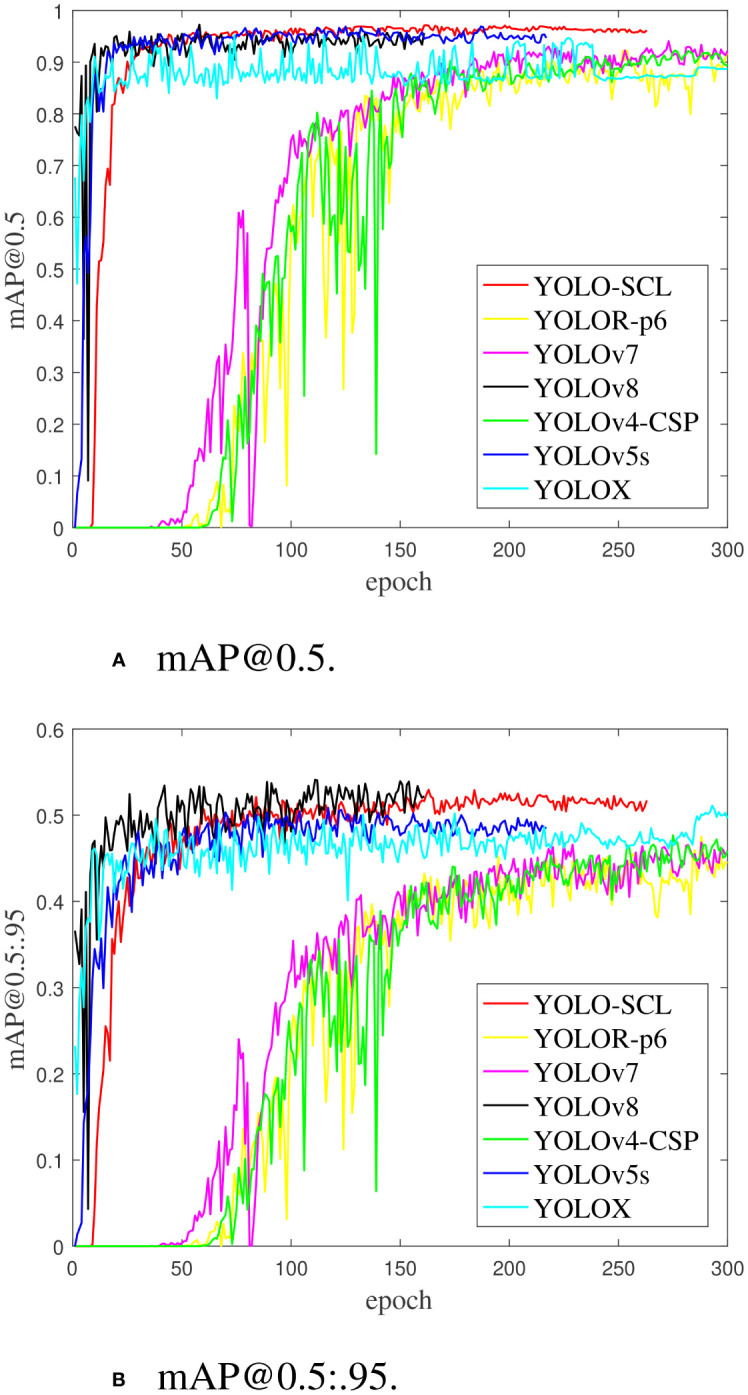
mAP@0.5 and mAP@0.5:.95 iteration curves of training results on different models. **(A)** mAP@0.5. **(B)** mAP@0.5:.95.

**Figure 8** shows the iterative curves of mAP@0.5 and mAP@0.5:.95 during the training process of the YOLO series of detection models. The mAP@0.5 and mAP@0.5:.95 curves of the YOLOv5s, YOLOX, and proposed YOLO-SCL models almost intersect at the 50th epoch. The detection accuracy of the YOLOv5s and YOLOX models rapidly improves at the early stage. In contrast, the YOLO-SCL model steadily improves at the later stage and achieves results superior to those of the YOLOv5s, YOLOX and YOLOv8 models. The mAP@0.5 and mAP@0.5:.95 curves of the YOLOv7, YOLOv4-CSP, and YOLOR-p6 models are considerably inferior to that of the proposed YOLO-SCL model. For these three models, the curves fluctuate more with poor robustness than that of the proposed model.

**Figure 9:**
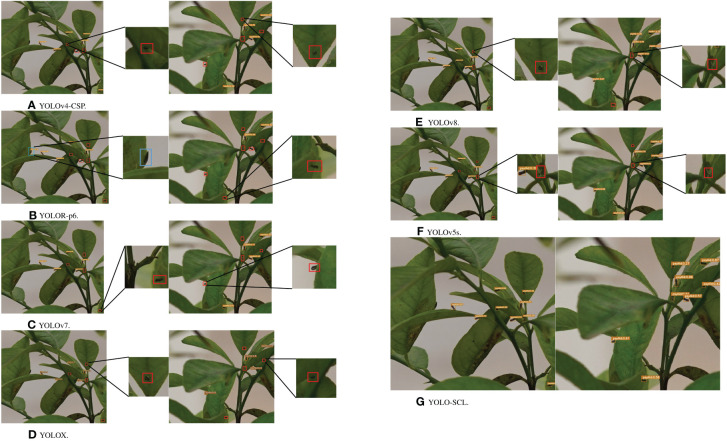
Test results of different models on the test images. The test results from top to bottom are YOLOv4-CSP, YOLOR-p6, YOLOv7, YOLOX, YOLOv8, YOLOv5s, and YOLO-SCL. Red boxes indicate missed detections, and blue boxes indicate false detections. **(A)** YOLOv4-CSP. **(B)** YOLOR-p6. **(C)** YOLOv7. **(D)** YOLOX. **(E)** YOLOv8. **(F)** YOLOv5s. **(G)** YOLO-SCL.

**Figure 9** shows the results of the YOLO series of detection models. The proposed YOLO-SCL model demonstrated effective detection of a higher number of citrus psyllids in complex backgrounds than that of other models, thereby improving detection accuracy. Under the conditions of low light and blurred individual psyllid due to filming problems, the improved model exhibited accurate identification with a low leakage rate, indicating that the *ɡ_n_* Conv ECA module enhances the model’s ability to express features in critical areas, making it highly efficient in extracting target features in complex backgrounds. Meanwhile, the lightweight C3 module retains the feature information of small targets to a certain extent, contributing to better target localization and identification of the model, thereby reducing the number of missed and false citrus psyllids detections.

### 5.5 Hyperparameter optimization of the detection model based on BWOA

In this study, 25 hyperparameter combinations of the YOLO-SCL model were used as the optimization objects. Based on the optimization iterative mechanism of swarm intelligence, the characteristics of BWOA, such as few control parameters and fast optimization rate, were used to map the hyperparameters into individual feasible solutions of BWOA and obtain the optimal hyperparameter combinations that are suitable for citrus psyllid detection tasks. The control parameters included 50 epochs, 100 iteration numbers, and 10 iteration numbers. The hyperparameter optimization results are shown in **Tables 5** and **Table 6** compares the detection results before and after optimization. **Table 6** shows that before and after the optimization of hyperparameters, the precision, recall, mAP@0.5, and mAP@0.5:.95 were improved by 1.44%, 1.60%, 0.11%, and 0.75%, respectively, indicating that the optimization of hyperparameters can effectively improve the precision of the detection of citrus psyllids, and further improve the performance of the model.

**Table 5:** Results before and after hyperparameter optimization.

Hyperparameter	Before Optimization	After Optimization	(lb, ub)
Lr0	0.001	0.04185	(0.00005, 0.1)
Lrf	0.01	0.14641	(0.01, 1.0)
Momentum	0.937	0.70022	(0.6,0. 98)
Weight decay	0.0005	0.00014	(0.0, 0.001)
Warmup epochs	3.0	1.16490	(0.0, 5.0)
Warmup momentum	0.8	0.07027	(0.0, 0.95)
Warmup bias lr	0.1	0.01957	(0.0, 0.2)
Box	0.05	0.02334	(0.02, 0.2)
Cls	0.5	0.63575	(0.2, 4.0)
Cls pw	1.0	0.58352	(0.5, 2.0)
Obj	1.0	1.59100	(0.2, 4.0)
Obj pw	1.0	0.58352	(0.5, 2.0)
Anchor t	4.0	3.64620	(2.0, 8.0)
Hsv h	0.015	0.02919	(0.0, 0.1)
Hsv s	0.7	0.03530	(0.0, 0.9)
Hsv v	0.4	0.01103	(0.0, 0.9)
Degrees	0.0	0.05874	(0.0, 45.0)
Translate	0.1	0.29687	(0.0, 0.9)
Scale	0.5	0.01554	(0.0, 0.9)
Shear	0.0	3.26980	(0.0, 10.0)
Flipud	0.0	0.13026	(0.0, 1.0)
Mosaic	1.0	0.02767	(0.0, 1.0)
Mixup	0.0	0.09442	(0.0, 1.0)
Copy pasts	0.0	0.21605	(0.0, 1.0)
anchors	–	2.40950	(2.0, 10.0)

**Table 6:** Comparison of detection results before and after hyperparameter optimization.

	P(%)	R(%)	mAP@0.5(%)	mAP@0.5:.95(%)
Before Optimization	93.73	92.35	97.07	53.43
After Optimization	95.17	93.95	97.18	54.18

**Figure 10:**
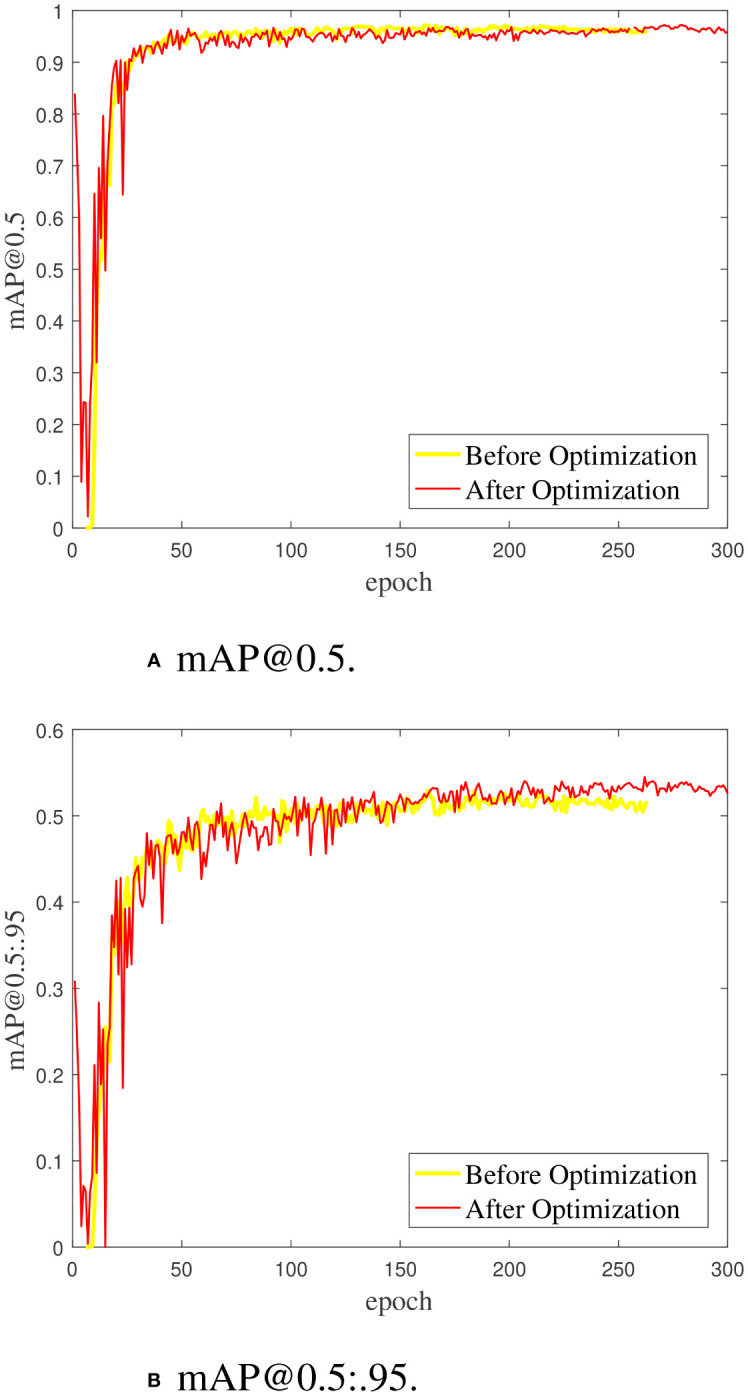
The mAP@0.5 and mAP@0.5.95 curves of the YOLO-SCL model training process before and after hyperparameter optimization. **(A)** mAP@0.5. **(B)** mAP@0.5:.95.

**Figure 10** shows the iterative curves of mAP@0.5 and mAP@0.5:.95 of the YOLO-SCL model training process before and after optimization. The mAP@0.5 and mAP@0.5:.95 curves of the optimized model in the first 10 epochs have large fluctuations, indicating that the optimization of the hyperparameter combination affects the stability of the detection model. However, the iterative curve gradually stabilized as the epoch increased, and better detection accuracy was obtained.

### 5.6 Lightweight C3 module grad-cam analysis

In this study, lightweight experiments are performed on the C3 modules in layers 17, 20, and 23 of the YOLOv5s model neck network, and the results are shown in **Table 7**. The YOLOv5s model lightened for the 17th layer C3 module has 0.48% lower mAP@0.5 and 0.50% lower mAP@0.5:.95 than the baseline model YOLOv5s, and this lightning operation affects the performance of the baseline model. The mAP@0.5 of the YOLOv5s model lightened for the 20th and 23rd C3 modules are 0.68% and 0.82% higher than that of the YOLOv5s model, respectively, while the mAP@0.5:.95 of the 20th lightened YOLOv5s model are 1.63% higher than that of the 23rd lightened YOLOv5s model. Therefore, in this study, we chose to improve the 21st layer C3 module of the YOLO-SCL model.

**Table 7:** Experimental results of lightweight C3 modules at layers 17, 20, and 23 in the neck network of the YOLOv5s model.

Methods	mAP@0.5 (%)	mAP@0.5:.95 (%)
YOLOv5s+Light C3(17)	95.41	50.28
YOLOv5s+Light C3(20)	96.57	52.31
YOLOv5s+Light C3(23)	96.71	50.68

**Figure 11:**
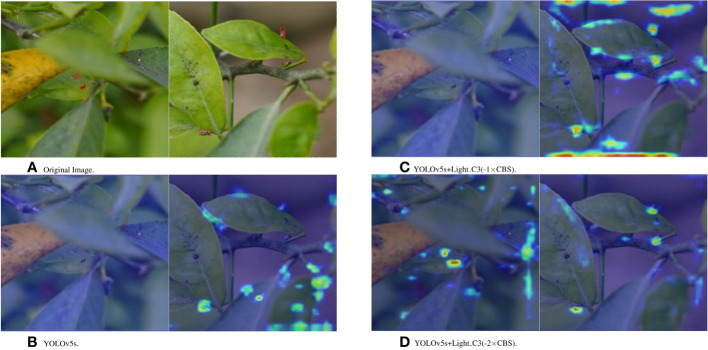
Grad-CAM thermograms of the YOLOv5s+Light C3 model trained on the citrus psyllid dataset and YOLOv5s model layer 20. **(A)** Original Picture. **(B)** YOLOv5s. **(C)** YOLOv5s+Light C3(-1×CBS). **(D)**YOLOv5s+Light C3(-2×CBS).

To further verify that the lightweight C3 module can reduce the loss of detailed feature information of small targets by deleting the convolutional layers, this study uses the Grad-CAM method to analyze the attention focused on the target with different improvements in the 20th layer of the C3 module. **Figure 11** shows the Grad-CAM thermograms of the several YOLOv5s+Light C3 models trained on the citrus psyllid dataset and the 20th layer of the YOLOv5s model. Figs. b(left), c(left) and d(left) show that the YOLOv5s and YOLOv5s+Light C3(-1×CBS) models lose the feature information of the target to a certain extent, which affects the model’s performance of detecting the target, whereas the YOLOv5s+Light C3(-2×CBS) model not only retains the feature information of the target but also improves the attention to the target. Figs. b(right), c(right), and d(right) show that the YOLOv5s and YOLOv5s+Light C3(-1×CBS) models exhibit a more diffuse attention area and are more affected by the complex environment, whereas the YOLOv5s+Light C3(-2×CBS) model focuses more on the target. The comprehensive experimental results show that by lightening the C3 module and deleting the convolutional layer, the feature information of the tiny target is retained to the maximum extent, and the attention is more focused on the target, improving the detection accuracy of the target.

### 5.7 Detection model porting deployment based on NVIDIA JETSON AGX XAVIER embedded platform

In this study, the YOLO-SCL model porting deployment was performed using NVIDIA Jetson AGX Xavier edge computing platform based on the ARM architecture. The Jetson AGX Xavier edge computing platform is 100 × 87 mm in size, has a GPU of 512 cores, a CPU of 8 cores, and 32 GB of storage. The system of this platform is Ubuntu 18.04.

**Figure 12:**
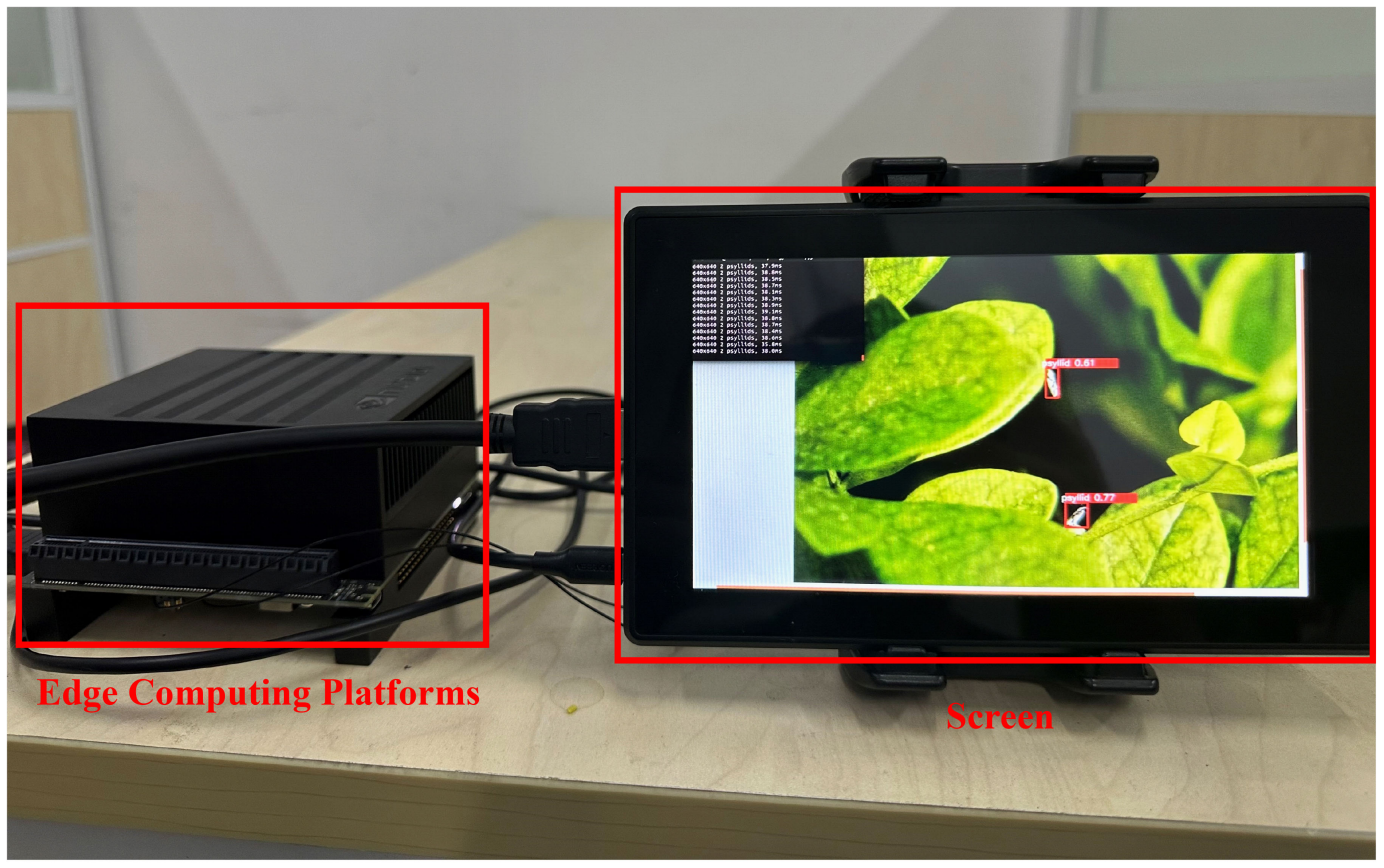
Jetson AGX Xavier edge computing platform model porting deployment detection results.

To facilitate the porting of the YOLO-SCL model, a runtime environment was configured on a Jetson AGX Xavier to debug the hardware performance of the platform, specifically the deep learning gas pedal engine. The model porting and deployment results are shown in **Figure 12**, demonstrating that the average processing time of a single-frame image is 38.8 ms, and the power consumption is 16.85 W.

## 6 Conclusions and analyses

### 6.1 Discussion

The mAP@0.5 results of the YOLO-SCL model on the citrus psyllids detection task were better than those of the other six target detection models, from the prediction result graphs, the YOLO-SCL model effectively solved the missed and false problems that occurred in the other six models. The above results show that the improved architecture combining the *ɡ_n_* Conv and lightweight C3 modules effectively improves the feature representation of the model for target regions. In the follow-up work, attempts will be made to apply the improved approach to different target detection models for citrus psyllid detection tasks as well as to apply the YOLO-SCL model to more tasks in other fields.

### 6.2 Conclusions

In this study, a YOLO-SCL model for detecting citrus psyllids in natural environments was proposed to improve the detection accuracy of citrus psyllids in response to problems such as the difficulty of accurately detecting small targets and complex background interference. First, the *ɡ_n_* Conv module based on spatial channel interaction was proposed to improve the model’s detection accuracy for small targets; the module is realized through the combination of global spatial and local channel interactions. Second, to maximize the retention of small target feature information, the 21st layer of the C3 module in the YOLO-SCL model was lightened and improved, making the model more target-aware. In addition, optimization of the hyperparameters in the YOLO-SCL model using BWOA. The main conclusions are as follows:

- The YOLO-SCL model achieved 97.07% mAP@0.5 and 53.43% mAP@0.5:.95 with a model parameter count and computation of 6.92 M and 15.5 GFlops, respectively. The model considerably improved the detection of citrus psyllids and was ported and deployed on the edge computing platform with an average processing time of 38.8 ms and power consumption of 16.85 W for a single-frame image. In addition, optimization of the hyperparameters in the YOLO-SCL model using BWOA resulted in higher mAP@0.5 and mAP@0.5:.95 of 97.18% and 54.18%, respectively.
- Compared with the other six detection models, the YOLO-SCL model achieved the highest mAP@0.5. The YOLO-SCL model is 6.92 M and 15.5 GFlops, which are 14.25% and 2.52% lower than those of the conventional YOLOv5s model, respectively. The above data show that the improved model has achieved better performance in recognition accuracy and efficiency.
- This study discusses citrus psyllids detection in natural environments; the above results show that the YOLO-SCL model performs well on the citrus psyllid detection task and provides some values for studying different small target detection tasks.

## Data availability statement

The original contributions presented in the study are included in the article/supplementary material, further inquiries can be directed to the corresponding author/s.

## Author contributions

SL: Conceptualization, Funding acquisition, Methodology, Supervision, Validation, Writing – original draft. XZ: Writing – original draft, Data curation, Formal Analysis, Methodology, Software, Visualization, Resources. ZL: Conceptualization, Funding acquisition, Supervision, Validation, Writing – review & editing. XL: Data curation, Formal Analysis, Writing – review & editing. YC: Data curation, Formal Analysis, Writing – review & editing. WZ: Data curation, Formal Analysis, Writing – review & editing.
